# Optimization of the Culture Medium Composition to Improve the Production of Hyoscyamine in Elicited *Datura stramonium* L. Hairy Roots Using the Response Surface Methodology (RSM)

**DOI:** 10.3390/ijms11114726

**Published:** 2010-11-18

**Authors:** Amdoun Ryad, Khelifi Lakhdar, Khelifi-Slaoui Majda, Amroune Samia, Asch Mark, Assaf-Ducrocq Corinne, Gontier Eric

**Affiliations:** 1 Laboratoire des Ressources Génétiques et Biotechnologie, Ecole Nationale Supérieure Agronomique, 16200 El-Harrach-Alger, Algeria; E-Mails: r_amdoun@yahoo.fr (A.R.); m.khelifi@ensa.dz (K.-S.M.); samia_amr@hotmail.fr (A.S.); 2 Université de Picardie Jules Verne, UFR of Sciences, LAMFA, CNRS-UMR 6140, 33 rue Saint Leu, 80039 Amiens cedex 1, France; E-Mail: mark.asch@u-picardie.fr; 3 Research Unit EA3900 BioPI-UPJV Biologie des plantes et contrôle des insectes ravageurs, Université de Picardie Jules Verne, UFR of Sciences, Ilot des poulies, 33 rue Saint Leu, 80039 Amiens cedex 1, France; E-Mails: corinne.assaf@u-picardie.fr (A.-D.C.); eric.gontier@u-picardie.fr (G.E.)

**Keywords:** *Datura stramonium*, hyoscyamine, hairy root, medium components, optimization, Response Surface Methodology

## Abstract

Traditionally, optimization in biological analyses has been carried out by monitoring the influence of one factor at a time; this technique is called *one-variable-at-a-time*. The disadvantage of this technique is that it does not include any interactive effects among the variables studied and requires a large number of experiments. Therefore, in recent years, the Response Surface Methodology (RSM) has become the most popular optimization method. It is an effective mathematical and statistical technique which has been widely used in optimization studies with minimal experimental trials where interactive factors may be involved. This present study follows on from our previous work, where RSM was used to optimize the B5 medium composition in [NO^3−^], [Ca^2+^] and sucrose to attain the best production of hyoscyamine (HS) from the hairy roots (HRs) of *Datura stramonium* elicited by Jasmonic Acid (JA). The present paper focuses on the use of the RSM in biological studies, such as plant material, to establish a predictive model with the planning of experiments, analysis of the model, diagnostics and adjustment for the accuracy of the model. With the RSM, only 20 experiments were necessary to determine optimal concentrations. The model could be employed to carry out interpolations and predict the response to elicitation. Applying this model, the optimization of the HS level was 212.7% for the elicited HRs of *Datura stramonium*, cultured in B5-OP medium (optimized), in comparison with elicited HRs cultured in B5 medium (control). The optimal concentrations, under experimental conditions, were determined to be: 79.1 mM [NO^3−^], 11.4 mM [Ca^2+^] and 42.9 mg/L of sucrose.

## 1. Introduction

Hairy roots (HRs) are an efficient system for the production of secondary metabolites [1–4]. However, the performance of this production system depends on the composition of the nutrient medium, which not only affects the content of secondary metabolites [5–11], but also the response to elicitation [12]. The main aim of optimization is to improve the performance of the system by increasing production at a low cost [13,14].

In general, the experimental procedure of optimization is achieved by studying a single factor at any one time. While this factor is modified to find the optimal response, others are kept at a constant level. This is known as the *one-variable-at-a-time-technique*. Clearly, its major disadvantage is that interactions among the factors are not considered so it does not reflect all the potential effects on the process [15]. Another drawback is the large number of experiments needed, requiring additional time and expense. More efficient analytical techniques are based on Response Surface Methodology (RSM) [14]. This was first proposed by Box and his collaborators in 1951 [16] as a method to determine the optimal conditions which maximize or minimize a response. It enables a large amount of data to be obtained from a reduced number of experiments, including the potential interactions between the studied factors [13]. The RSM can be defined as a group of statistical and technical tools used to study the relationship between a response of interest and several input variables. The model has to describe the behavior of a group of data with a view to making statistical predictions. The aim is the simultaneous optimization of several factors to lead to the best performance of a particular system [14].

During recent years, the RSM has been used extensively for optimization in many areas of industrial research and process development in chemistry and biochemistry. Although Calam [17] was the first to suggest the application of RSM in biotechnology, its field was limited [18].

Its use for the optimization and analysis of biotechnological processes with microorganisms and enzymatic engineering has given good results. For example, the work of Maddox and Richert [19] in optimizing bacterial media and Cheynier *et al.* [20] in optimizing enzymatic activity can be cited. However, the application of this analytical technique to systems with plant tissues has been limited.

This study follows the work of Amdoun *et al.* [12] and presents two main aims:

- First, to use the RSM method for the optimization of nutrients (nitrate, calcium and sucrose) in the culture medium B5 [Gamborg, 1968] to improve hyoscyamine production in elicited HRs;
- Second, to show the value of the RSM method in the optimization of several responses in plant material cultures.

## 2. Materials and Methods

### 2.1. Plant Materials

The HRs, obtained by genetic transformation and selection, were subcultured in the same conditions as described previously [12].

### 2.2. Elicitation

The use of the desirability function enabled the Jasmonic Acid concentration (JAC) and the exposure time (ET) to be optimized. The optimal JA concentration of 0.06 mM with an exposure time of 24 h was the optimal compromise. The elicitation treatment was initiated 24 h before harvesting the HRs line and was removed for analysis on the 28th day of culture. This length of time was identified as the best stage for elicitation for the highest hyoscyamine content [12].

The solution of Jasmonic Acid (JA) [(−)-jasmonic acid from Sigma-Aldrich] was prepared by dissolving Jasmonic Acid in an adequate volume of ethanol. The solution was filtered through membrane filter (pore size: 0.2 mm Nalgene) and completed with sterile distilled water. The HRs were elicited with this stock solution at 0.06 mM.

The controls were the same HRs lines in the same conditions of culture but without elicitation (ethanol-water solution without Jasmonic Acid). All cultures were conducted in Petri dishes containing 20 mL of B5 medium, in darkness at 26 ±1 °C.

### 2.3. Extraction and Hyoscyamine Analysis

Alkaloids were extracted using a method described by Amdoun *et al.* [12]. Hyoscyamine was analyzed using the GC-MS method previously reported by Kartal *et al.* [21].

### 2.4. Theory of RSM

The response surface methodology (RSM) consists of an adjustment of empirical models to the data obtained experimentally. Linear (1st degree) or quadratic (2nd degree) mathematical models are employed to describe the system to be optimized [22]. When several factors influence a particular system, it is impossible to screen and to control the contribution of each factor so only those factors which have a major influence must be considered. The screening design, such as the factorial designs 2^k^, can be used to meet this objective. They are efficient and economical [15].

To evaluate the form of the true response, a second degree model is used. The factorial designs 2^k^ are used to determine the first-order effects but these are inefficient when additional effects, like the second-order effects, are so important. Experimental central points in the factorial design 2^k^ can be added to evaluate the shape. The polynomial function must contain other factors, which include the interaction between the various experimental variables. To determine a critical point (maximum, minimum or saddle), the polynomial function of second degree must contain quadratic factors.

The model of second degree is given by equation (1), where *Y* is the response, *α*_0_ is the intercept of the *y* axis, *α_j_*, *α_jj_*,…, *α_jl_* are the various coefficients of the model (linear and quadratic), *X_j_* and *X_l_* are the independent variables (factors) and *ε* is the error of model with ∀*i*, *V*(*ε_i_*) = *σ*^2^ (homoscedasticity) and ε ∼ *N* (0, *σ*^2^) (normal distribution).

(1) $$Y=\alpha_{0}+\sum_{j=1}^{k}\alpha_{j}X_{j}+\sum_{j=1}^{k}\alpha_{jj}X_{j}^{2}+\sum_{j<1}\sum_{l=2}^{k}\alpha_{jL}X_{j}X_{l}+ɛ$$

The equation system (1) can be resolved by the Method of Least Squares (MLS) and may be written in matrix form (2) [23], where *y* is the (*n*, 1) vector of measured responses, *X* is the (*n*, *p*) matrix of the mathematical model of factor levels, and *α* is the (*p*, 1) vector of unknown coefficients.

(2) $$\begin{array}{cccccc} \underset{y}{\underset{︸}{\left[\begin{array}{c} y_{1}\\ y_{2}\\ .\\ .\\ .\\ y_{n} \end{array}\right]}} & = & \underset{X}{\underset{︸}{\left[\begin{array}{cccccc} 1 & x_{11} & . & . & . & x_{1k}\\ 1 & x_{21} & . & . & . & x_{2k}\\ . & . & . & . & . & .\\ . & . & . & . & . & .\\ . & . & . & . & . & .\\ 1 & x_{n1} & x_{n2} & . & . & x_{nk} \end{array}\right]}} & \underset{\alpha }{\underset{︸}{\left[\begin{array}{c} \alpha_{0}\\ \alpha_{1}\\ .\\ .\\ .\\ \alpha_{k} \end{array}\right]}} & + & \underset{ɛ}{\underset{︸}{\left[\begin{array}{c} {ɛ}_{1}\\ {ɛ}_{2}\\ .\\ .\\ .\\ {ɛ}_{n} \end{array}\right]}} \end{array}$$

With *n* responses, and *y* and *p* coefficients in the model, there are *n* equations and *n* + *p* unknowns. To solve the system, the Method of Least Squares is used. After some calculations, the solution of system (2) is given by Equation (3):

(3) $$\hat{a}={\left({}^{t}XX\right)}^{-1}{}^{t}XY$$

The low and high levels of variables (factors) are denoted by −1 and 1, respectively. The levels of *X_i_* variable are coded and obtained from Equation (4), where *X_i_* is the independent variable coded value, *A_i_* is the independent variable real value, *A*_0_ is the independent variable real value on the center point and Δ*A_i_* is the step change value.

(4) $$X_{i}=\left(A_{i}-A_{0}\right)/\Delta A_{i}\;$$

The most used second-order symmetric designs for the RSM are: The general factorial design, the Box-Behnken design, the Central Composite Design and Doehlert’s design. These differ by the location of the experimental points in the studied region, the number of factor levels kept, the number of experiments and the blocks [14].

### 2.5. Global Predicted Capacity, Analysis and Diagnostic of the Model

When the relationship between the variables and the response has been established by the modeling, predictions can be made. However, the mathematical model obtained after adjustments to experimental results, sometimes cannot describe the studied domain. It is necessary to analyze and examine the diagnostic of the obtained model to evaluate its pertinence to describe the studied phenomena. If the analysis and the diagnostic are satisfactory, the defined model can be used in predictions, but only if the conditions are identical and the standard error is present.

The global predicted capacity of a mathematical model is generally explained by the coefficient of determination *R*^2^ A large value of this coefficient does not necessarily suggest that the model is a good one. The *R*^2^ value improves if the variables are high even if they are not statistically significant [24]. Because *R*^2^ alone is not a measure of the model’s accuracy, it is also necessary to take into account the value of the Absolute Average Deviation (AAD) [13]. The AAD is a direct method which describes deviations, so its value must be as small as possible between the measured and the predicted data [13].

During the analysis, the statistical global significance of the model is determined by variance analysis (ANOVA, Fisher’s Test: *F-*value). The lack of fit test is also applied to estimate the model (fitting). The statistical significance of the model terms is determined by the Student test (*t-*value). The model is adjusted to the experimental data when it is significant and when the lack of fit value of the model is not significant. The truth of the hypothesis related to the homoscedasticity and the normal distribution of variations is very important during the diagnostic. The visual analysis of graphical plots of deviations gives some information about the robustness of the model. Concerning the influential observations on the predictions of the model, the main statistics are the leverage, Cook’s distance and the DFFITS.

### 2.6. Determination of the Optimum

Depending on the object, the optimal point can be characterized as the maximum, the minimum or the saddle. The value of the maximal point can be calculated from the first derivative of the model’s equation. The positive values of the explanatory variables, where the first derivative is equal to zero, correspond to the optimal values. The accuracy of the values can be verified by comparing the predicted values obtained with the mathematical model, and the measured values obtained after the experiments with the same conditions.

### 2.7. Application of the RSM for Optimization of the Culture Medium B5

The aim of this paper is to optimize the composition of the medium, which influences the response to elicitation [12,25,26]. This work succeeds a precedent paper [12], where we applied the screening analysis to the influence of minerals on the hyoscyamine content of elicited HRs.

As has been reported in [12], nitrate [NO_3_^−^], calcium [Ca^2+^], the combination [NO_3_^−^ × Ca^2+^] and phosphorus [H_2_PO_4_^−^], are the most significant factors in the intensity of the response to elicitation for hyoscyamine production by HRs. Although phosphorus [H_2_PO_4_^−^] is statistically efficient, only [NO_3_^−^] and [Ca^2+^] are used for optimization by RSM. Sucrose is also included for optimization due to its effect on biomass and alkaloid production [6,8]. The minimal and maximal values of nitrate and calcium have been selected from the precedent results of Amdoun *et al.* [12]. The real values of the concentrations have been codified by the Equation (4) (see Section 2.4).

The Central Composite Design (CCD) is used to obtain the measured responses which will be useful for the mathematical model. The CCD was presented by Box and Wilson in the 1950s. It consists of the following two parts:

- A factorial design with at least one experimental point located in the center of the experimental area;
- A star design whose axial points *−α* and *+α* are located on the axis of each factor. This design is particularly adapted to the progressive acquisition of results with a factorial design 2^k^.

**Figure 1.**
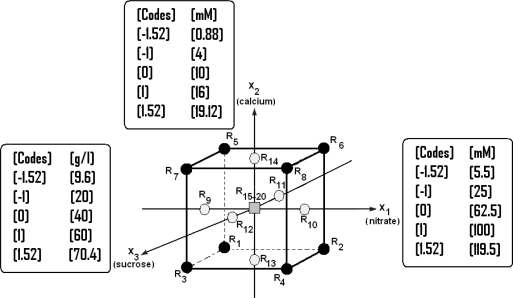
Experimental region and levels of each of the three factors of the CCD (*X_1_*: [NO_3_^−^], *X_2_*: [Ca^2+^] and *X_3_*: sucrose).

It is necessary to carry out the experiments corresponding to the star design’s points and to calculate whether the results are explained by the linear model. Figure 1 shows the studied experimental region, the range and the levels of the studied variables. Five levels have been considered for each of the three studied factors (NO_3_^−^, Ca^2+^ and sucrose). So, the application of CCD consists of carrying out 20 experiments combining three factors, six of which are located in the center of the studied region. The optimal criterion is orthogonality, in this case *α* calculated is equal to ±1.52 [27]. Each point (R_1_…R_20_) corresponds to one experiment. The points R_1_ to R_8_ represent the factorial design 2^k^. The points R_9_ to R_14_ are the star design. The points R_15_ to R_20_ represent the experiment done in the central experimental design.

For all experiments, the HRs cultures were performed in Petri dishes containing 20 mL of B5 medium [28]. In order to avoid other modifications of the medium due to the addition of other counterions, each element was chosen and then HNO_3_ for [NO_3_^−^] and Ca(OH)_2_ for [Ca^2+^] were added to the medium before adjustment of the pH to 5.8 [29]. The quantity of inoculum for each dish was 0.3 g fresh weight from the root tips of HRs of *Datura stramonium*. The measured response was the hyoscyamine level (mg/L). The averages were calculated from the three replicates. The control corresponds to the same HRs line; in the same B5 medium containing 25 mM [NO_3_^−^], 1.0 mM [Ca^2+^] and 3% sucrose; and the same conditions of temperature and darkness. Dry weight was obtained by oven drying the HRs for 48 h at 40 °C. The final dry biomass is expressed by the average of the three values with a precision balance.

## 3. Results and Discussion

### 3.1. Global Predictivity of the Model

The coefficient of determination *R*^2^ measures the variability explained by the factors and their interactions in the observed responses [30]. It is 0.97 for the model, from which it can be concluded that 97% of the HS level of elicited HRs is attributed to independent variables and only 3% of the total variability is not explained by the model. The AAD value is low (1.2%) and the value of deviation is σ = 0.9. Thus, the model explains the global variability; it is globally predictive.

### 3.2. Analysis of the Quadratic Model

The *F*-value and the value of probability show that the model is statistically significant. The lack of fit test is not significant (Table 1), which indicates that the model fits well with the experimental data.

All the linear terms related to [NO_3_^−^], [Ca^2+^], [NO_3_^−^/Ca^2+^] interaction and the sucrose effect are significantly positive (Table 2). The linear effect of the latter is almost identical to that of [Ca^2+^]. As the concentrations of these three elements increase in the B5 medium, the response to elicitation of HRs becomes more significant up to a certain limit where the response is reduced.

The beneficial effect of [NO_3_^−^] is twofold: it improves the biomass [7,8,12] and the HS production by HRs simultaneously [8,12]. It is one of the crucial elements of the medium to improve the level of HS in elicited HRs. Effectively, its deficiency during the first week of culture, negatively affects the response to elicitation of HRs [12]. Moreover, its excess in the medium (from 95 mM) shows a negative effect on the biomass [8,12,31]. The [Ca^2+^] is in second position for increasing the HS level after elicitation [12]. The [Ca^2+^] ion activates Putrescine Methyl Transferase (PMT), which is one of the enzymes involved in the biosynthesis of tropane alkaloids [5,9]. It is a secondary messenger and participates in signal transduction and cellular regulation. Its intracellular flux is activated by a stimulus (such as elicitation) and also induces the defense reactions [26,32]. For sucrose, Saenz-Carbonell and Loyola-Vargas [6] showed that it improves the specific production of hyoscyamine. In our case, sucrose particularly increases the biomass.

### 3.3. Diagnostic of the Model

#### 3.3.1. Diagnostic Plot

**Figure 2.**
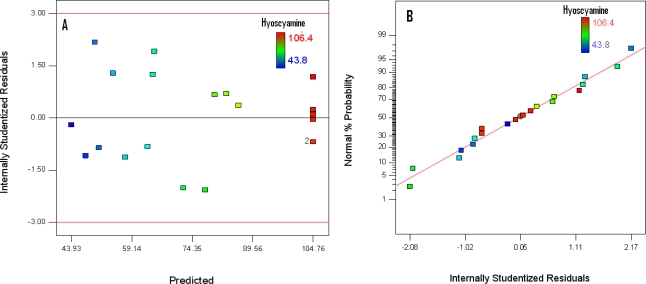
Diagnostic plot: (**A**) Studentized residuals *versus* predicted values; (**B**) normal probability plot of the studentized residuals.

The validity of a model can be evaluated by the residual analysis. We defined as residual, the difference between the observed values and the calculated values obtained by the model. It is the part which is not explained by the equation of the model. This analysis can also detect some outliers among the total data. We use principally graphical methods for the residual analysis, such as the graphical presentation of the residuals as a function of the estimated values [33]. This is essential to check the homoscedasticity hypothesis of the errors. If the selected model is adequate, the residuals are distributed uniformly on a horizontal band of the graph. The analysis of Figure 2A shows a random distribution of the residuals as a function of the predicted values, so the homoscedasticity hypothesis is verified. Another crucial hypothesis on the residual is the normal distribution. The graphical representation of the residuals is an important tool for diagnostics [34,35]. Figure 2B shows a normal distribution of the Studentized residuals, which are independent of each other.

#### 3.3.2. Influential Observations and Accommodation

If the value of an observation diverges from the supposed form of the distribution of the total observations, it is called an outlier. If this form is modified, the observation can converge with the new model [33]. After the detection of outlier observations, the accommodation can be done in different ways, for example by complementary data, re-specifying the model or removing observations [36]. An outlier value is called an influential observation if its presence in the data affects the estimated coefficients of the model [36]. The classical measurement of the influential observations on the predictions of a model are the leverage *h_i_*, Cook’s distance and DFFITS (Table 3).

We called the leverage *h_i_*, for an observation *i* as the estimation of the *i*^th^ variable response which is influenced by the value of the corresponding independent variable *X_i_*; *h_i_* is between 0 to 1 [33]. Table 3 does not show extreme values concerning *h_i_* for the global observations. Nevertheless, the values of the observations R_1_ to R_8_ are close to 1. The *h_i_* does not take into consideration the residual of the observation so, to complete the diagnostic, Cook’s distance and the DFFITS are examined. The latter are close to leverage and residuals. Cook’s distance of an observation is a measurement of the observation’s influence on the total predictions of the model. The value of Cook’s distance must be <1 [37]. The observations R_4_ and R_5_ show a main influence on the global predictions of the model (Table 3). The DFFITS measurement of an observation is the measurement of this observation’s influence on its value’s predictivity by the model. The DFFITS value could be less than 1 [38]. Table 3 shows that the experiments R_2_ to R_7_, R_9_, R_13_ and R_14_ have crucial influences. From this diagnostic, we expect deviations if we are looking at the predictions in the experimental domain near these points. To improve the model’s accuracy, the first operation is to select the terms which are most significant. In this case, the model can be written as:

(5) $${\hat{Y}}_{HS}=104.7+10.5X_{1}+5.5X_{2}+4.2X_{3}+3.5X_{1}X_{2}-16.4X_{1}^{2}-14.4X_{2}^{2}-14.5X_{3}^{2}\;$$

**Figure 3.**
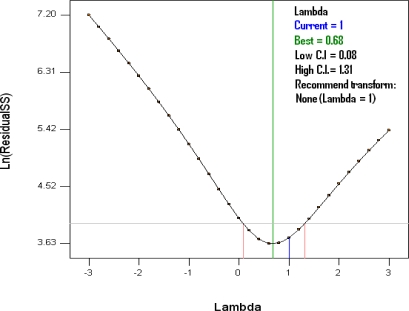
Box-Cox plot for power transformations (*Y*^λ^).

After this operation, only the observations R_4_, R_8_ and R_14_ presented main DFFITS values (Table 3). The data needed no transformation (*Y*^λ^), as confirmed by the Box-Cox Plot exam (Figure 3). We decided to adjust the outliers, which originate from measurement errors, by their removal. After the removal of the R_4_ observation, which has the highest DFFITS value, only the R_14_ observation shows an extreme value. After this elimination, the diagnostic of influential observations reveals no outliers. We can observe that the normal distribution of residuals and their independence, the homoscedasticity and the statistical significance of the model are still verified.

The removal of the observations R_4_ and R_14_ improves the accuracy of the estimated coefficients. The CV% goes from 2.0 to 0.9, in this case the AAD value was calculated as 0.4%. More precisely, it is the model *Ŷ_HS_* (Equation 6) which has been selected for the response surface methodology analysis and the determination of optimal concentrations.

(6) $${\hat{Y}}_{HS}=104.7+11.0X_{1}+6.0X_{2}+4.1X_{3}+4.3X_{1}X_{2}-16.4X_{1}^{2}-14.4X_{2}^{2}-13.7X_{3}^{2}\;$$

### 3.5. Response Surface (RS) Analysis and Determination of Optimal Concentrations

**Figure 4.**
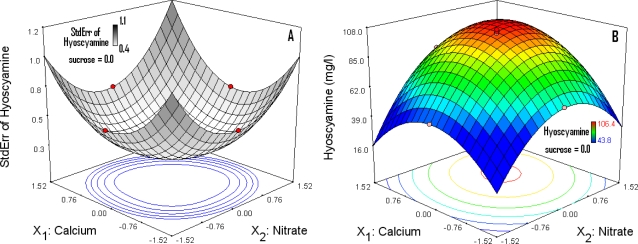
Response Surface plots showing effects of [NO_3_^−^] and [Ca^2+^] as a function of standard error (StdErr) (**A**) and the HS level for elicited HRs (**B**).

The graphical representation of the standard error (StdErr) function (Figure 4A) is symmetric and its highest value (>1) is obtained in the four corners of the experimental area. Nevertheless, its value is 0.3 inside the central area. This low value is related to a good model quality, so the model Equation 6 can be used to evaluate the values of the *Ŷ_HS_* response in the global experimental domain.

The RS is the graphical representation of the mathematical model in the experimental domain. Figure 4B shows the response surface for the HS level from elicited HRs as a function of [NO_3_^−^] (*X*_1_) and [Ca^2+^] (*X*_2_). The sucrose value has been fixed to its coded value 0.0 (equivalent to 40 mg/L). From this figure, for different combinations of [NO_3_^−^] and [Ca^2+^], the responses can be predicted. This is an effective tool to see the simultaneous effect of [NO_3_^−^] and [Ca^2+^] on the HS level from elicited HRs. The improvement in the HS level is correlated with the increasing [NO_3_^−^, Ca^2+^] concentrations until limits where the response decreases, beyond the central zone of the experimental domain (Figure 4B). The beneficial effect of the combination has been described by Amdoun *et al.* [12]. In the case of a low response with low [NO_3_^−^, Ca^2+^] values, the cells are in a critical condition for plant defense reactions [12]. The high [NO_3_^−^, Ca^2+^] values have a negative impact on the biomass and also on the HS level. Our results are identical with those obtained by Sikuli and Demeyer [7] for the biomass. These authors found low values of biomass and HS for HRs of *Datura stramonium* cultivated in B5 medium with high [NO_3_^−^, Ca^2+^] concentrations. This effect is greater when the [Ca^2+^] value is high. We also note that the nitrate reductase activity measured in the HRs is high in this medium [7].

All the combinations [NO_3_^−^, Ca^2+^] located in the red area (Figure 4B) illustrate the optimal conditions. Nevertheless, the local optimum concentrations correspond to positive values of variables where the first derivatives (δ*Y_HS_*/*x*_1_; δ*Y_HS_*/*x*_2_; δ*Y_HS_*/*x*_3_) of the model Equation 6 cancel each other out. The optimal concentrations in coded values are 0.37 for [NO_3_^−^], 0.26 for [Ca^2+^] and 0.14 for sucrose. This is equivalent to 79.1 mM, 11.4 mM and 42.9 g/L respectively in real values and corresponds to the optimized B5 medium (B5-OP). These optimal calculated concentrations are located in the area where the standard error function is lower and where the predicted accuracy of the model Equation 6 is confirmed.

According to the simulation of the HS level from elicited HRs, cultured in B5-OP medium, the predicted value is 107.90 mg/L. The calculated prediction interval, at 95%, is *PI* = [106.17; 109.72]. The measured level for the same concentrations and in the same conditions is 110.3 ±1.4 mg/L. This value is in the 95% *PI* range and validates the optimal concentrations of [NO_3_^−^], [Ca^2+^] and sucrose.

### 3.6. Optimization Level

**Figure 5.**
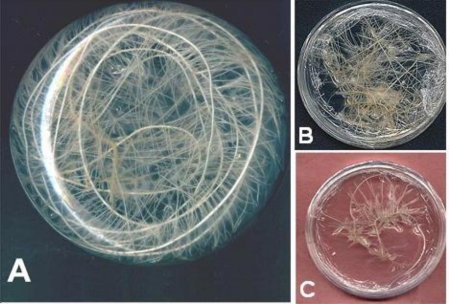
Appearance of HRs on the 28th day of culture in B5-OP (**A**) in 250 mL Erlenmeyer; (**B**) in Petri dishes and in B5 control (**C**) in Petri dishes.

Figures 5A and 5B show a culture of HRs in B5-OP medium in a Petri dish or Erlenmeyer. It displays a high biomass in comparison with HRs cultivated in B5 control (Figure 5C).

There is a significant improvement in biomass (51.2%) with the B5-OP medium in comparison with the HRs control (Table 4). A significant difference was observed for the specific production of HS (mg/g DW) in HRs with or without elicitation. The optimization was determined as 81% and 101.2% for elicited or non-elicited HRs respectively in B5-OP medium.

Finally, the improvement in the level of HS (mg/L) was 173.6% for non-elicited HRs, and 212% for elicited HRs cultivated in B5-OP, in comparison with the control medium.

## 4. Conclusion

The selection of significant statistical variables enables the accuracy of the model to be improved. After the diagnostics, some outliers related to the model with the global terms are eliminated after the screening. This selection of outliers must be carried out carefully; their removal is implemented when they are due to pure error. In our case, only two observations were removed after the study of the screening of variables.

Optimization of the B5 medium improves the response to elicitation. Under optimal conditions, the optimization of the HS level is 212.7% for elicited *Datura stramonium* HRs cultivated in B5-OP medium, in comparison with HRs in the B5 control.

The optimal concentrations for the selected line and in our conditions are: 79.1 mM, 11.4 mM and 42.9 g/L for [NO_3_^−^], [Ca^2+^] and sucrose, respectively. These values correspond to the solutions obtained with the first derivatives of the model. Nevertheless, all the concentrations included in the optimal area of the surface response can be used.

This new approach to the optimization of B5 medium could be employed to maximize the response to elicitation. The B5-OP medium significantly influenced the biomass and alkaloid production. This was possible by the simulation and the predictability of the quadratic model used. Indeed, unlike classical methods, this mathematical model is not time consuming and a large number of experiments are not needed to optimize the parameters. Mathematical modeling is a powerful tool for biological studies. In this work, by applying the RSM, only 20 experiments were required to optimize the B5 medium composition in terms of [NO_3_^−^], [Ca^2+^] and sucrose. This quadratic model led to a demonstration of the effects of these nutrients on the HS level of HRs elicited by Jasmonic Acid (JA).

However, the HS level of the elicited HRs, cultured in B5-OP medium, can still be improved by an irregular deficiency in [NO_3_^−^] [12]. The feasibility needs to be confirmed and the time and the frequency need to be determined. For production in a bioreactor, it is clear that the optimal concentrations must be added according to the B5 medium volume and the biomass.

## Figures and Tables

**Table 1.** ANOVA table for the quadratic model (results in bold are significant).

**Source**	**Sum of Squares**	**df**	**Mean Square**	***F*-value**	***p*-value**
Model	9537.1	10	953.7	414.1	**0.0**
Residual	20.7	9	2.3		

Lack of fit	16.1	4	4.0	4.3	0.1
Pure Error	4.7	5	0.9		

Total	9557.8	19			

**Table 2.** Analysis terms for the quadratic model (results in bold are significant).

**Model Terms**	**Coefficient Estimate**	**t-statistic**	***p*-value**
Intercept	104.8	172.2	-
*x*_1_: nitrate	10.5	24.6	**0.0**
*x*_2_: calcium	5.5	12.9	**0.0**
*x*_3_: sucrose	4.2	9.8	**0.0**
*x*_1_*x*_2_	3.5	6.5	**0.0**
*x*_1_*x*_3_	1.0	1.9	0.1
*x*_2_*x*_3_	1.0	1.9	0.1
*x*_1_^2^	−16.4	−35.4	**0.0**
*x*_2_^2^	−14.4	−31.1	**0.0**
*x*_3_^2^	−14.5	−31.3	**0.0**
*x*_1_*x*_2_*x*_3_	0.7	1.3	0.2

**Table 3.** Diagnostics for influential observations (Results in bold are outliers).

**Model with Full Terms**									

**Run Order (*n*)**	**Variable Code Levels**			**Measured**	**Predicted**	**Residual**	***h_i_* Leverage**	**Cook’s Distance**	**DFFITS**
	*x*_1_ [NO_3_^−^]	*x*_2_ [Ca^2+^]	*x*_3_ [sucrose]						
R_1_	−1	−1	−1	43.8	43.9	−0.1	0.8	0.0	−0.4
R_2_	1	−1	−1	56.8	57.5	−0.7	0.8	0.5	**−2.5**
R_3_	−1	1	−1	46.8	47.5	−0.7	0.8	0.5	**−2.4**
R_4_	1	1	−1	70.8	72.0	−1.3	0.8	**1.7**	**−5.6**
R_5_	−1	−1	1	51.2	49.8	1.4	0.8	**2.0**	**6.5**
R_6_	1	−1	1	65.2	64.4	0.8	0.8	0.7	**2.8**
R_7_	−1	1	1	55.2	54.4	0.8	0.8	0.7	**2.9**
R_8_	1	1	1	86.2	86.0	0.2	0.8	0.1	0.7
R_9_	−1.52	0	0	50.0	50.9	−0.9	0.6	0.1	**−1.0**
R_10_	1.52	0	0	83.6	83.0	0.7	0.6	0.1	0.8
R_11_	0	−1.52	0	62.3	63.1	−0.8	0.6	0.1	−0.9
R_12_	0	1.52	0	80.6	79.9	0.7	0.6	0.1	0.7
R_13_	0	0	−1.52	66.7	64.8	1.8	0.6	0.4	**2.7**
R_14_	0	0	1.52	75.5	77.5	−2.0	0.6	0.5	**−3.1**
R_15_	0	0	0	103.8	104. 8	−1.0	0.2	0.0	−0.3
R_16_	0	0	0	104.7	104.8	−0.0	0.2	0.0	0.0
R_17_	0	0	0	105.1	104.8	0.3	0.2	0.0	0.1
R_18_	0	0	0	106.4	104.8	1.6	0.2	0.0	0.5
R_19_	0	0	0	103.8	104.8	−1.0	0.2	0.0	−0.3
R_20_	0	0	0	104.9	104.8	0.1	0.2	0.0	0.0

**Model with Only Significant Terms (Equation 5)**									

R_4_	1	1	−1	70.8	74.8	−4.0	0.6	0.9	**−4.7**
R_8_	1	1	1	86.2	83.2	2.3	0.5	0.5	**2.4**
R_14_	0	0	1.52	75.5	77.5	−2.0	0.6	0.5	**−2.2**

**Table 4.** Biomass and HS production of HRs cultivated in B5 control medium or B5-OP medium after the 28th day of culture

		**Biomass (g DW/L)**	**Production of HS**			

			**(mg/g DW)**		**(mg/L)**	

			**Without Elicitation**	**With Elicitation**	**Without Elicitation**	**With Elicitation**
B5 *		8.4 ± 0.6	2.1 ± 0.1	4.2 ± 0.6	17.6 ± 1.6	35.3 ± 2.0
B5-OP **		12.7 ± 0.2	3.8 ± 0.1	8.5 ± 0.3	48.3 ± 2.3	110.3 ± 1.4
Optimization		51.2%	81%	101.2%	173.6%	212.7%

LSD test	differences	−4.3	−1.7	−4.3	−30.6	−75.0

	±limits	0.9	0.2	0.8	4.4	13.8

	significance	significant	significant	significant	significant	significant

*: B5 control (25 mM NO_3_^−^, 1.0 mM Ca^2+^ and 3% sucrose);

**: B5-OP optimized (79.1 mM NO_3_^−^, 11.4 mM Ca^2+^ and 42.9% sucrose).
